# Fibrin Sealant Derived from Human Plasma as a Scaffold for Bone Grafts Associated with Photobiomodulation Therapy

**DOI:** 10.3390/ijms20071761

**Published:** 2019-04-10

**Authors:** Karina Torres Pomini, Daniela Vieira Buchaim, Jesus Carlos Andreo, Marcelie Priscila de Oliveira Rosso, Bruna Botteon Della Coletta, Íris Jasmin Santos German, Ana Carolina Cestari Biguetti, André Luis Shinohara, Geraldo Marco Rosa Júnior, João Vitor Tadashi Cosin Shindo, Murilo Priori Alcalde, Marco Antônio Hungaro Duarte, Daniel de Bortoli Teixeira, Rogério Leone Buchaim

**Affiliations:** 1 Department of Biological Sciences (Anatomy), Bauru School of Dentistry, University of São Paulo (USP), Bauru 17012-901, Brazil; karinatorrespomini@gmail.com (K.T.P.); danibuchaim@usp.br (D.V.B.); jcandreo@usp.br (J.C.A.); marcelierosso@usp.br (M.P.d.O.R.); brucoletta@hotmail.com (B.B.D.C.); anacarolinacb25@gmail.com (A.C.C.B.); andreshinohara@yahoo.com.br (A.L.S.); jvshindo@gmail.com (J.V.T.C.S.); 2 Department of Human Morphophysiology, Medical and Dentistry School, University of Marilia (UNIMAR), Marília 17525-902, Brazil; daniel.dbt@hotmail.com; 3 Department of Human Anatomy and Neuroanatomy, Medical School, University Center of Adamantina (UniFAI), Adamantina 17800-000, Brazil; 4 Department of Dentistry, Faculty of Health Science, Universidad Iberoamericana (UNIBE), Santo Domingo 10203, Dominic Republic; irish_knaan@hotmail.com; 5 Department of Health Science, University of the Sacred Heart (USC), Bauru 17011-160, Brazil; geraldomrjr@yahoo.com.br (G.M.R.J.); murilo_alcalde@hotmail.com (M.P.A.); 6 Department of Anatomy, University of the Ninth of July (UNINOVE), Bauru 17011-102, Brazil; 7 Department of Dentistry, Endodontics and Dental Materials, Bauru School of Dentistry, University of São Paulo (USP), Bauru 17012-901, Brazil; mhungaro@fob.usp.br

**Keywords:** bone regeneration, bone repair, fibrin sealant, biomaterial, photobiomodulation therapy, low-level laser therapy

## Abstract

Fibrin sealants derived from human blood can be used in tissue engineering to assist in the repair of bone defects. The objective of this study was to evaluate the support system formed by a xenograft fibrin sealant associated with photobiomodulation therapy of critical defects in rat calvaria. Thirty-six rats were divided into four groups: BC (*n* = 8), defect filled with blood clot; FSB (*n* = 10), filled with fibrin sealant and xenograft; BC^PBMT^ (*n* = 8), blood clot and photobiomodulation; FSB^PBMT^ (*n* = 10), fibrin sealant, xenograft, and photobiomodulation. The animals were killed after 14 and 42 days. In the histological and microtomographic analysis, new bone formation was observed in all groups, limited to the defect margins, and without complete wound closure. In the FSB group, bone formation increased between periods (4.3 ± 0.46 to 6.01 ± 0.32), yet with lower volume density when compared to the FSB^PBMT^ (5.6 ± 0.45 to 10.64 ± 0.97) group. It was concluded that the support system formed by the xenograft fibrin sealant associated with the photobiomodulation therapy protocol had a positive effect on the bone repair process.

## 1. Introduction

There are currently available treatment options for the repair of bone defects, but their effectiveness is limited in large defects, and the influence of extrinsic factors such as smoking and alcohol exposure are unfavourable in this process [1,2,3]. Annually more than two million bone grafts are performed worldwide, the second most frequent tissue transplantation, being surpassed only by blood transfusion [4,5].

Among all available types, the autologous graft is still considered the gold standard, since all the necessary properties in bone regeneration in terms of osteoconduction, osteoinduction, and osteogenesis are combined [6,7]. However, its availability is limited, and morbidity at the donor site has led to the development of new bone substitutes that restore, ameliorate, or prevent aggravation of compromised tissue function [8,9].

In order to solve this problem, tissue engineering has developed xenografts that are skeletal derivatives of other species, mainly bovine, with satisfactory osteoconductive properties and widely used in reconstructive procedures with greater scientific evidence among biomaterials [4,10].

To form a graft material mouldable to the surgical bed, facilitate its insertion and agglutination, and prevent its dispersion and collapse of soft tissue into the defect, biodegradable polymers known as scaffolds are used as three-dimensional supports for the lodging of cells and biologically active molecules, providing a favourable environment for tissue regeneration [10].

Among the scaffolds, fibrin sealants derived from human blood may have the potential to guide this process of bone remodelling, because it has compatible physiological characteristics to human tissue and thus is readily colonised by the surrounding cells. Thus, they allow surgeons to influence and improve the cellular microenvironment in vitro or in vivo, increasing the success rate of the bone graft [11].

Other attempts have been studied to minimise the time of bone healing and to reduce the chance of possible complications arising from the abnormal regeneration process. Among them, low-intensity pulsed ultrasound [12] and laser photobiomodulation therapy have been highlighted by their satisfactory effects on bone metabolism and repair, due to their possible osteogenic effect [13,14].

Laser photobiomodulation therapy is a non-invasive treatment method with relatively low cost [15]. However, there are controversies regarding the best parameters to be used to obtain an effective result in the process of bone repair of critical size defects filled with biomaterials [5].

Despite the growing interest in blood-derived biomaterials in the reconstruction of bone defects, in the literature reviewed, no studies were found on the effects of the combination of sealant with bone grafts and alternative methods such as photobiomodulation. Thus, this study evaluated the support system formed by a xenograft fibrin sealant associated with the protocol of photobiomodulation therapy in critical size defects in rats.

## 2. Results

### 2.1. Microtomographic Analysis

**Figure 1:**
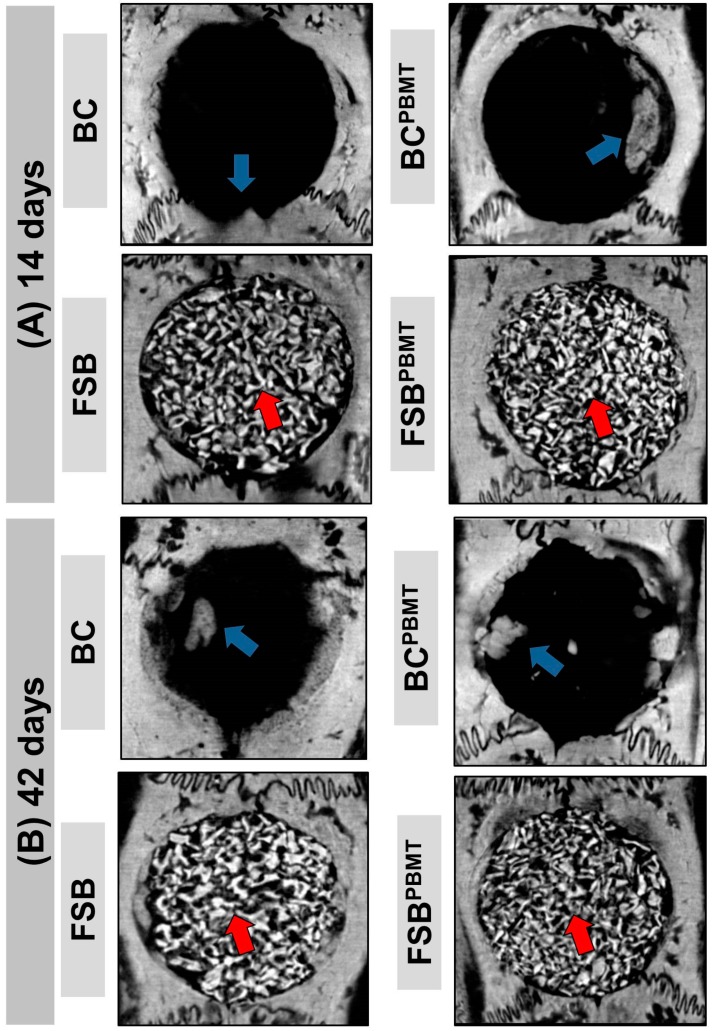
Microtomographic images showing the evolution of the repair of defects filled with clot and fibrin sealant plus xenograft (biomaterial) with or without low-level laser biostimulation therapy. Biomaterial particles (red arrow) and newly formed bone tissue (blue arrow). Two-dimensional trans-axial cuts at (**A**) 14 days; and (**B**) 42 days, respectively.

In microtomographic images, at 14 days, it was observed that in all the defects, the new bone formation occurred centripetally from the critical defect margins towards the centre. All groups exhibited a continuous increase of new bone formation during the analysed periods; however, in no specimen was there a complete closure of the defect, and the formed bone was restricted to the defect borders (blue arrow—Figure 1A).

In the groups where the defects were filled with fibrin sealant associated with the xenogeneic graft (FSB—Fibrin sealant with xenograft, and FSB^PBMT^—Fibrin sealant with xenograft and photobiomodulation therapy), the images showed the surgical cavity filled with the materials implanted and fine bone trabeculae adjacent to the border of the defect and under the dura mater. In animals that were biostimulated with a low-level laser, a more evident formation of the bone tissue was observed in FSB^PBMT^ compared to the FSB group (Figure 1A).

In the subsequent period, at 42 days, an increase in the amount of bone tissue, interweaving the biomaterial, in a more organised configuration, especially in the FSB^PBMT^ group was observed. The xenograft particles were still evident (red arrow), with some areas of remodelled tissue at the defect margins (Figure 1B).

### 2.2. Histological Evaluation

In all groups, the repair of bone defects occurred centripetally, with the absence of necrotic tissue, the presence of bone cells, osteoid matrix, and budding of new blood vessels at the site.

**Figure 2:**
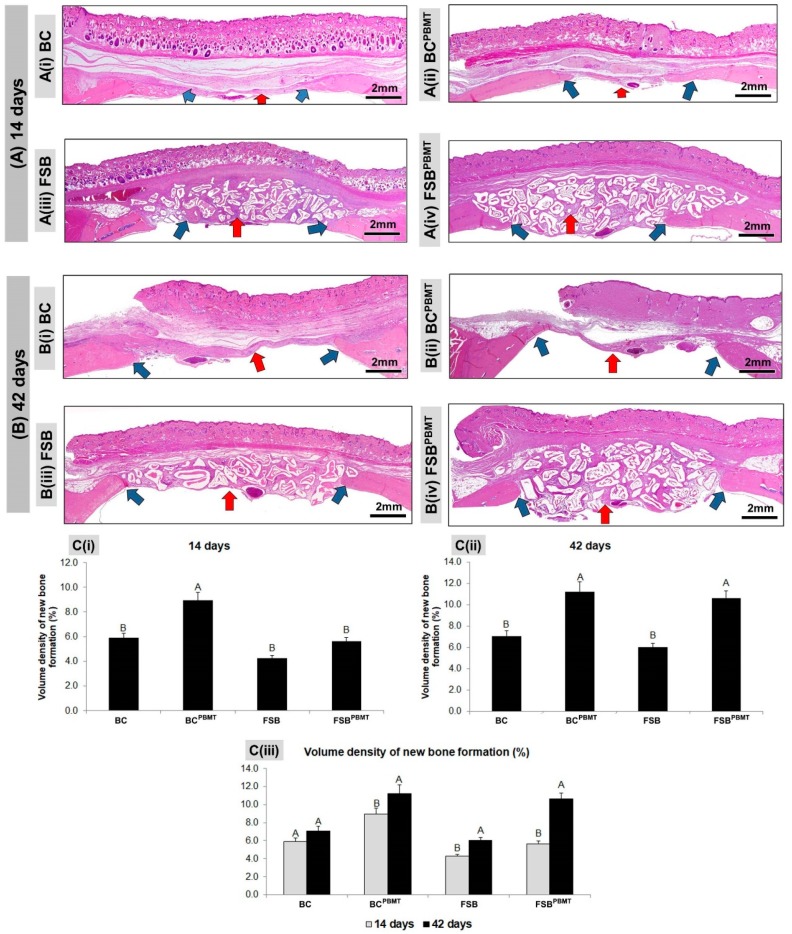
Panoramic histological views at (**A**) 14 days; and (**B**) 42 days, respectively; (**C**) graphs of volume density of newly formed bone in skull defects filled with a blood clot or fibrin sealant plus xenograft and with or without laser photobiomodulation therapy. (**A**) A(i)–A(ii) bone formation (blue arrows) occurring at the defect border and under the dura mater surface. A(iii)–A(iv): the defect showed trabecular bone formation (blue arrows) adjacent to the defect border, in a more advanced stage of bone maturation. (**B**) B(i)–B(ii) both groups showed similar bone formation limited to the defect border and a large region filled with fibrous connective tissue (red arrows); B(iii)–B(iv) a large part of the defect was filled by connective tissue and biomaterials (red arrows), but in the FSB^PBMT^ group, greater bone formation defect could be observed compared to the FSB group; (**C**) Graphs of newly formed bone showed smaller bone formation in the non-biostimulated group (BC and FSB) than the biostimulated group (BC^PBMT^ and FSB^PBMT^). (BC and BC^PBMT^: *N* = 4/group and periods), (FSB and FSB^PBMT^: *N* = 5/group and periods). C(i) and C(ii) where different letters (A≠B) indicate a statistically significant difference between groups in the same period and C(iii) where the different letters (A≠B) indicate a statistically significant difference in the same group in the two periods analysed (*p* < 0.05). (HE; original magnification × 4; bar = 2 mm).

**Figure 3:**
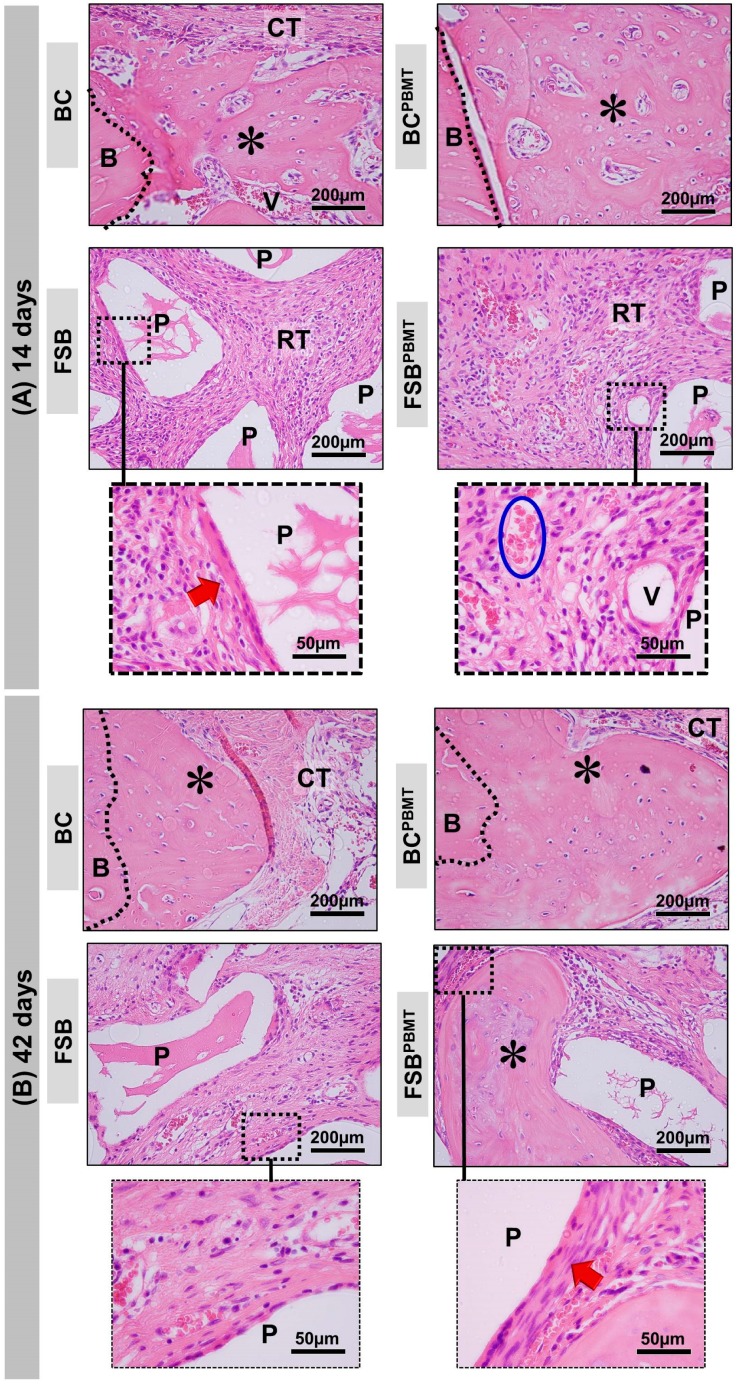
Details of the evolution of the bone healing of the skull defects filled with a blood clot or fibrin sealant plus xenograft (biomaterial) with or without low-level laser biostimulation therapy. (**A**) At 14 days, BC and BC^PBMT^: the defect shows the trabecular bone formation (asterisks) adjacent to the defect border and spaces between the trabeculae filled by connective tissue. FSB: the defect was filled by particles of the biomaterial (P) surrounded by connective tissue with some inflammatory cells (RT—reactional tissue). Collagen fibres surrounding the xenograft particles (red arrow). FSB^PBMT^: the defect was filled by particles of the biomaterial (P) surrounded by connective tissue with some inflammatory cells (RT—reactional tissue). Presence of many red blood cells (inside the blue lined area) and blood vessels (V) permeating connective tissue; (**B**) At 42 days, BC and BC^PBMT^: the new bone shows a gradual increase in thickness of the trabeculae leading to a compact structure. FSB: the new bone formation increases, becoming compact, there is a presence of xenograft particles and a decrease in inflammatory response. In FSB^PBMT^, the collagen fibres are arranged in more layers surrounding the particles. (HE; original magnification × 40; bar = 200 µm; and Insets, magnified images × 100; bar = 50 µm).

At 14 days, all BC (defect filled with Blood Clot without photobiomodulation therapy) and BC^PBMT^ (defect filled with Blood Clot with photobiomodulation therapy) animals presented incomplete bone repair both in the height and in the conformation of the newly formed bone, which was irregular along the dura mater (Figure 2A(i)–A(ii) and Figure 3A). In the animals in the BC group, the central area of the defect was predominantly filled by loose connective tissue with small loci of new bone formation at the defect border, but in the BC^PBMT^ animals, the defects were filled by immature bone and more obvious blood vessels (Figure 2A(i)–A(ii) and Figure 3A).

In all animals of the FSB and FSB^PBMT^ groups, in the same experimental period, the defects presented with large amounts of the biomaterial. The new bone formation also occurred from the defect border, with trabecular conformation, being more pronounced in the FSB^PBMT^ group. The presence of inflammatory infiltrate was identified in both groups, diffusely distributed in the interstitial space (Figure 2A(iii)–A(iv) and Figure 3A).

At 42 days, in the BC group, the formed connective tissue filled the entire extent of the defect, maintaining a seemingly smaller thickness in relation to the remaining (original) bone, and the new bone formed was limited to the proximities of the injured borders. In the BC^PBMT^ animals, biostimulated with low power laser, the defect was still filled by a large amount of connective tissue, exhibiting a thin layer of bone tissue (asterisk) with diploe characteristics, and in some cases partial closure of the defect, but without recovery of its height (Figure 2B(i)–B(ii) and Figure 3B).

In the same period, in all FSB and FSB^PBMT^ animals, the surgical area was almost completely filled by biomaterial particles, without any significant changes in relation to the previous period. The bone formation remained limited to the edges, but with a denser and lamellar arrangement. The tissue reaction appeared to be in the resolution phase, with the most fibrotic interstitial space. In the FSB^PBMT^ group, the reduction of oedema was more evident resulting in the formation of a denser stroma with more cells and with concentric collagen fibres forming a capsule around the biomaterial (Figure 2B(iii)–(iv) and Figure 3B).

### 2.3. Histomorphometric Evaluation

At 14 days of the repair process and after a quantitative evaluation of the volume density of the newly formed bone, it was observed that animals of the BC^PBMT^ group presented the highest means (8.9 ± 0.64) with significant difference in relation to the other experimental groups BC, FSB, and FSB^PBMT^ (5.9 ± 0.38; 4.3 ± 0.46; 5.6 ± 0.45, respectively), that were not significantly different to each other (Figure 2C(i)).

In the 42-day period, the groups biostimulated with a low-power laser (BC^PBMT^ and FSB^PBMT^) presented the highest means, but without significant difference between them (11.22 ± 0.94; 10.64 ± 0.97, respectively). However, the animals in the aforementioned groups showed a significant difference when compared to the non-biostimulated animals BC and FSB (7.06 ± 0.49; 6.02 ± 0.32, respectively), but did not show any significant difference when compared to each other (Figure 2C(ii)).

The evaluation of the volume density of the newly formed bone within the same group in the two experimental periods (14 and 42 days) revealed that bone formation was higher in all groups in the 42-day period, with a significant difference between periods except for the BC group (Figure 2C(iii)).

## 3. Discussion

The existing scientific evidence in the field of tissue engineering indicates promising results in the treatment of bone defects with the use of fibrin sealants derived from human plasma as scaffolds for cellular development [11,16,17,18,19]. However, there is no data in the literature which reports its effect on the bone repair process when associated with xenograft and alternative therapeutic methods. Thus, the results in this study show that the association of these treatments favoured the repair process of critical bone defects in the calvaria of rats.

The bone calvarium defect rat model is the most used among others in the scientific literature since it provides a clinically relevant evaluation of regenerative therapies and bone substitute materials, allowing for more effective clinical interventions. The defect produced is perfectly reproducible, fast, and does not require fixation for stabilisation, as compared to long bones [20].

The search for noninvasive methods, such as low intensity ultrasound (LIPUS), electromagnetic fields, and laser photobiomodulation therapy, has been increasing exponentially in recent years to improve the bone healing process [12]. As a consequence, there are numerous clinical and experimental studies with low-level laser photobiomodulation therapies, but so far without consensus on the optimal parameters for the bone repair process [5].

This study used a wavelength of 830 nm, a power density of 258.6 mW/cm^2^, and mode of continuous operation, corroborating previous studies that presented satisfactory results in the process of bone repair [21].

Therefore, with the knowledge that the biomodulation effects of the laser are intrinsically related to the wavelength and that the loss of intensity may compromise its function, the right choice of the spectral band has become of extreme importance in the treatment. Thus, the wavelength in the infrared spectrum became widely used due to its lower loss, which can reach up to 37% of its intensity after a depth of 2 mm [22]. Knowing previously that the pre-calvarial tissue thickness in the rat has small dimensions, it is assumed that the loss is minimal. In situations exceeding 2 mm, there may be a maximum loss of 162.92 mW for each cm^2^ of tissue, with the same protocol used in this study.

In addition, the infrared spectrum, between 780 and 1100 nm, is based on non-thermal mechanisms, which do not generate a significant increase in tissue temperature (up to 37.5 °C). In excitation states, a fraction of energy is converted into heat, which causes local and transient increases in the temperature of absorbent chromophores, without heating the total cell [23].

To evaluate the potential of fibrin sealants derived from human blood, this study comprised microtomographic, histological, and histomorphometric analyses. In the microtomographic analysis, at 14 days it was possible to observe the formation of new bone at the margins of the surgical wound in all groups, probably via the stimulation of growth factors released after craniotomy. The growth remained limited to this region until the end of the experiment, as reported in other experimental studies [24,25].

In the groups where the defects were filled with the fibrin sealant associated with the xenogeneic graft (FSB and FSB^PBMT^), the particles remained at the site of implantation without dispersion, corroborating with studies that report on the mechanical stability and the binding effects provided by the fibrin sealant to bone grafts [26,27].

Histologically, at 14 days, the BC and BC^PBMT^ groups exhibited new bone formed in the defect margins, overlapping the dura, with a trabecular and immature arrangement. This can be attributed to the action of growth factors in this region after vascular rupture due to craniotomy and the presence of the underlying periosteum, which is the main source of osteoprogenitor cells and osteoinductive factors [28]. At 42 days, the newly formed bone became lamellar and compact. These findings are generally observed in repair procedures in lesions similar to those performed in this study [3,29,30]. However, none of the defects presented complete closure, with a large part being filled by fibrous connective tissue, in agreement with studies that reported that this is a critical defect according to Gosain et al. [31], An et al. [32], and Maciel et al. [30].

In the two analysed periods, the animals biostimulated with the laser presented greater evidence of new bone formation and greater tissue organisation at the end of the experiment [33]. These results are consistent with the literature that indicates the positive photobiomodulatory effects of the laser in the initial phases of bone repair, when, among several events, there is a proliferation of osteoblasts and differentiation of mesenchymal cells [34,35].

The defects filled with fibrin and xenograft (FSB and FSB^PBMT^) sealers showed intense angiogenesis as early as 14 days, as well as the presence of reactional tissue at 42 days of resolution [36]. The tissue reaction observed in these groups did not trigger a foreign body type granulomatous reaction, which suggests that the grafts used were biocompatible [26,37,38] and the biological response was consistent with the inflammatory process after implantation of the biomaterial [14,35,39,40,41,42,43].

Histomorphometric analysis of the clot-treated and biostimulated laser defects, BC^PBMT^, revealed a gradual and significant increase in bone volume during the experimental periods (8.9 ± 0.64 to 11.22 ± 0.94) in relation to the animals of group BC (5.9 ± 0.38 to 7.06 ± 0.49) in periods of 14 to 42 days, respectively [44]. The biological mechanisms involved in improving the growth of bone tissue irradiated by a low-power laser are still not clearly understood. Studies suggest that laser energy can excite intracellular chromophores, especially the cytochromes of mitochondria, stimulating the cellular activity and consequently increasing ATP concentration, calcium, protein synthesis, and signalling pathways actively interconnected with the differentiation of stem cells into osteoblasts [45,46].

In the group with defects treated with sealant and xenograft, FSB, the bone formation was increasing between the periods (4.3 ± 0.46 to 6.01 ± 0.32), but in lower volume density compared to the animals of the FSB^PBMT^ group (5.6 ± 0.45 to 10.64 ± 0.97), supporting the positive influence of laser photobiomodulation in the repair process. Similar results were reported by De Oliveira et al. [21] in calvarial defects of autogenous graft-filled rats treated with low-power laser, in which a higher bone formation was also observed in all analysed periods.

The results obtained in this experiment provide evidence that defects filled with fibrin sealant and xenograft, and treated with low-power laser presented an evolution in the tissue repair process, with a better response compared to the other groups investigated, suggesting that there was a photobiomodulatory action in the inflammatory process, with a more organised deposition of collagen fibres in the defect area and consequently with a more homogenous bone conformation.

### 3.1. Strengths

The present research is a pioneer experimental study on the use of a fibrin sealant derived from human blood and xenograft associated with the protocol of photobiomodulation therapy with the use of low-power laser demonstrating effective repair of nerve and bone lesions. In addition, the association provided ease of insertion, local haemostasis, and maintenance of the implanted materials in the surgical bed, allowing the accomplishment of procedures in a shorter operative time.

### 3.2. Limitations

One limitation of this study is the absence of a quantitative evaluation of the microtomographic images due to the similar radiopacity between the newly formed bone and the xenograft, which makes it difficult to quantify [47].

For prospective studies requiring repair of bone defects, analysis of other fibrin sealants may be proposed, such as a promising fibrin biopolymer free of human blood components [16], and associations with complementary therapies that present osteogenic potential as pulsed ultrasound (LUPUS) and ultralaser [12].

## 4. Materials and Methods

### 4.1. Blood-Derived Biomaterials—Fibrin Sealant

Tisseel Lyo™ (Baxter Healthcare Ltd., Norfolk, United Kingdom; Ministry of Health Registration n^o^: 1.0683.0182) is a two-component fibrin sealant that contains two of the proteins that make the blood clot, fibrinogen and thrombin. Tisseel Lyo is prepared as two solutions which mix when applied. When prepared, 1 mL of each solution contains human fibrinogen (as a clotting protein), 91 mg/mL in 3000 UIC/mL protein; aprotinin (synthetic) and human thrombin, 500 UI/mL, in 40 µmol/mL calcium chloride.

Initially, the vials containing lyophilised sealer protein concentrate and aprotinin solution, lyophilised human thrombin, and calcium chloride solution were preheated for approximately three minutes in a water bath at a temperature of 33–37 °C, with the aid of a mercury thermometer (Termometros Labor™, São Paulo, Brazil). Thereafter, the sealant protein concentrate was dissolved with the aprotinin solution to form the sealant solution. Simultaneously, the lyophilised human thrombin was dissolved with the calcium chloride solution to form the thrombin solution. The two solutions were kept in the water bath until use.

### 4.2. Biomaterial—Xenograft

The commercial demineralised bovine bone matrix (Bio-Oss™; Geistlich Pharma AG, Wolhusen, Switzerland; Ministry of Health Registration n^o^: 806.969.30002) is a natural biomaterial available as granules of cancellous bone (0.25–1 mm granule size; 2.0 g vial). The highly purified osteoconductive mineral structure is produced from natural bone in a multi-stage purification process and sterilisation is carried out by γ-irradiation. Thus, it is chemically as well as structurally comparable to the mineralized human bone. Bio-Oss™ contains pores of different sizes: macropores (300–1500 μm), micropores (size of Haversian and vascular marrow canals), and intracrystalline spaces (3–26 nm), resulting in an overall porosity of 70–75% and a wide internal surface area of almost 100 m^2^/g [48].

### 4.3. Experimental Design

Thirty-six adult male Wistar rats (Rattus norvegicus), 90 days old, weighing around 400 g, were obtained from the animal laboratory of the Ribeirão Preto campus of the University of São Paulo.

The animals were housed in conventional cages initially containing four animals each (alteration according to the animal weight recommended by the Animal Laboratory of Bauru School of Dentistry—University of São Paulo), with feeders and drinkers “*ad libitum*” (irradiated feed—Nuvilab rodents and filtered water), in an air-conditioned environment, air exhaustion, light-dark period 12L/12D, temperature 22 °C ± 2 °C, humidity 60% ± 10, lighting 150lux/1 m floor, maximum noise 70 dB (decibel—SPL, Sound Pressure Level). All experimental procedures in the animals were conducted with the approval of the Institutional Review Board in Animal Studies of the Bauru School of Dentistry, University of São Paulo (Protocol: CEEPA-019/2016).

**Figure 4:**
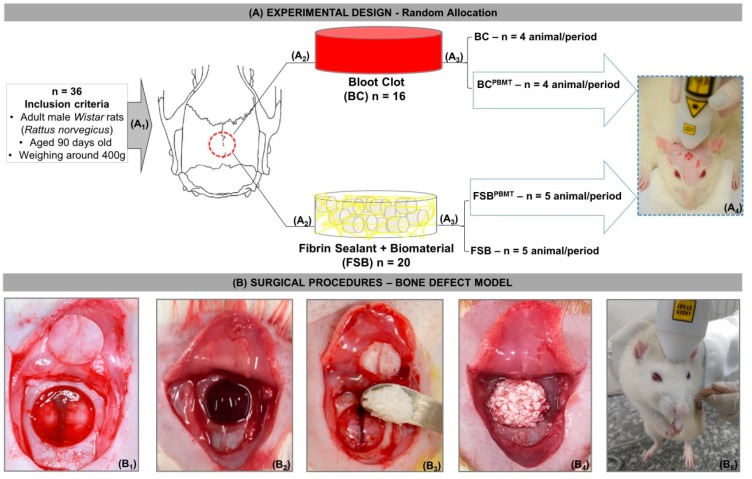
(**A**) Experimental design (**A_1_**) Random allocation: Thirty-six rats were divided into two groups; (**A_2_**) BC (*n* = 16)—Blood Clot and FSB (*n* = 20)—Fibrin Sealant + Biomaterial; (**A_3_**) After surgical procedures, two subgroups were preformatted according to treatment: BC, *n* = 8 (Blood Clot, the defect was filled with blood clot and without photobiomodulation), BC^PBMT^, *n* = 8 (Blood Clot, the defect was filled with blood clot and photobiomodulation), FSB, *n* = 10 (the defect was filled with a mixture of biomaterial and fibrin sealant and without photobiomodulation), and FSB^PBMT^, *n* = 10 (the defect was filled with a mixture of biomaterial and fibrin sealant and photobiomodulation); (**A_4_**) illustration of the four points of cross-application of the low-level laser on rat calvarium; (**B**) Surgical procedures—bone defect model; (**B_1_**) Osteotomy using an 8 mm trephine bur with exposure of the fragment removed from the parietal bones; (**B_2_**) defect filled with blood clot; (**B_3_**) deposition of the mixture fibrin sealant + biomaterial in the defect; (B_4_) defect filled with mixture; (**B_5_**) low-level laser therapy (photobiostimulation).

Initially, the animals were randomly divided into two groups: BC, *n* = 16 (Blood Clot, the defect was filled with a blood clot) and FSB, *n* = 20 (the defect was filled with a mixture of xenograft and fibrin sealant). After the surgical procedures, four subgroups were preformatted according to the treatment: BC, *n* = 8 (the defect was filled with blood clot without photobiomodulation), BC^PBMT^, n = 8 (the defect was filled with blood clot and photobiomodulation), FSB, *n* = 10 (the defect was filled with a mixture of fibrin sealant and biomaterial without photobiomodulation) and FSB^PBMT^, *n* = 10 (the defect was filled with a mixture of fibrin sealant and biomaterial and photobiomodulation) (Figure 4A).

### 4.4. Surgical Procedures

All surgical procedures were performed at the Mesoscopic Laboratory—discipline of Anatomy (Bauru School of Dentistry, University of São Paulo, Brazil) by the same team of professionals.

The animal surgeries were performed under general anaesthesia with an intramuscular injection of Ketamine (50 mg/kg i.m. (Dopalen ™, Ceva, Paulínia, SP, Brazil) and Xylazine (10 mg/kg i.m. (Anasedan ™, Ceva, Paulínia, SP, Brazil) followed by fronto-parietal trichotomy and disinfection with 10% povidone-iodine (PI). With a scalpel blade n. 10, a half-moon incision was made in the cranial tegument and folded to expose the calvarium. Then, a defect was created in the centre of the parietal bone using an 8 mm diameter trephine bur, under continuous irrigation with saline, exposing the dura mater [20,49] (Figure 4B_1_). The defects in the BC group were filled with 0.25 mm^3^ of cardiac puncture blood [50] (Figure 4B_2_).

In the FSB group, the defects were filled with 0.1 mm^3^ of xenograft incorporated into 40 µL of the reconstituted fibrinogen solution in aprotinin and 40 µL of reconstituted human thrombin solution in sodium chloride (proportion 1:1, according to the manufacturer’s recommendations) (Figure 4B_3_–B_4_). The amounts of xenograft and fibrin sealant used were previously established in a pilot study.

The periosteum and tegument were repositioned and sutured with nylon 5-0 (Mononylon^TM^, Somerville S.A, NJ, USA) and silk 4-0 (Ethicon^TM^ Johnson & Johnson Company, New Orleans, LA, USA), respectively, to provide stability to the graft, decreasing the risk of soft tissue collapse [20,51].

The postoperative care consisted of a single oral administration of acetaminophen at a dose of 200 mg/kg (Paracetamol, Medley, São Paulo, Brazil) dissolved in water, available in the cages.

### 4.5. Photobiomodulation Therapy Protocol 

The animals of Groups BC^PBMT^ and FSB^PBMT^ underwent laser irradiation (Laserpulse IBRAMED, Amparo, SP, Brazil) with continuous pulse GaAlAs (gallium–aluminium–arsenide). The following parameters were used for photobiomodulation therapy [21]—Table 1 below (Figure 4A_4_,B_5_):

### 4.6. Collection of Samples and Histological Procedures

All animals were killed with an overdose of a Ketamine/Xylazine mixture following the guidelines of the Brazilian College of Animal Experimentation after 14 and 42 days, BC and BC^PBMT^ groups—4 animals/period and FSB and FSB^PBMT^—5 animals/period. The cranial vaults with the lining skin were collected and fixed in 10% phosphate-buffered formalin for 48 h, and later, for examination in the microtomography.

### 4.7. MicroCT Scan (μ-CT)

The specimens were subjected to an X-ray beam scan in the computed microtomograph machine SkyScan 1174v2 (μ-CT—Bruker microCT, Kontich, Belgium). Initially they were packaged in an acrylic, cylindrical sample holder, (diameter 18.3 mm; height 10.9 mm), with exo- and endocranial aspects of the parietal bones in the vertical position. The images were captured with 13.76 µm voxel, 0.73° at each pace, and further reconstructed using the NRecon^®^ v.1.6.8.0, SkyScan, 2011, Bruker microCT, with the same reconstruction parameters for all specimens. Then, the reconstructed images were realigned using the DataViewer^®^ 1.4.4.0 software.

### 4.8. Histotechnical Processing

The specimens were washed in tap water for 24 h and immersed in 10% ethylenediaminetetraacetic acid (EDTA—a solution containing 4.13% Titriplex™ III Merck KGaA, Darmstadt, Germany and 0.44% sodium hydroxide Labsynth, São Paulo, Brazil), for a period of approximately 60 days [52]. Then, the collected bone fragments underwent successive standard histological staging and were finally included in Histosec^TM^ (Merck KGaA, Darmstadt, Germany). Semi-serial coronal cuts of 5 µm thickness were performed, prioritising the centre of the circular defect and stained with haematoxylin-eosin.

### 4.9. Histological and Histomorphometric Evaluation of Defects Bone Healing

The histological sections were analysed by light microscopy (Olympus model BX50) at approximate magnifications of × 4, 10, and × 40 in the Histology Laboratory of the Bauru School of Dentistry, the University of São Paulo (São Paulo, Brazil). To standardise and avoid bias, a training session was performed with an experienced pathologist.

Histological analysis of sections stained with HE consisted of an evaluative description of the healing events such as inflammation, granulation tissue, new bone formation, and remodelling, and the interaction among the biomaterial bone graft and the newly formed bone.

For morphometric evaluation, two central sections stained with HE were used for quantification of newly formed bone areas using an image capture system (DP Controller 3.2.1.276—2001–2006, Olympus Corporation, Tokyo, Japan). Initially, the total area to be analysed was established as every area of the surgical defect. The limits of this area were determined from the external and internal surfaces of the original calvarium on the right and left margins of the surgical defect. Then, the drawn lines were connected following their respective curvatures. Considering the total length of the defect, its centre point, measuring from this point, was 4 mm to the right and left to the edges of the surgical wound to determine the limits of the original surgical defect.

Morphometric analysis under light microscopy allowed the determination of the volumetric density (%), defined as the volume fraction of the entire graft filled by a given component/structure (newly formed bone). The volume density (Vvi) that is equal to the area density (AAi) was determined by AxioVision Rel. 4.8 Ink (Carl Zeiss MicroImaging GmbH, Jena, Deutschland), Vvi = AAi [53]. The area of graft filled by each structure (Ai) and the total area examined (A) were determined in pixels, and the volume density (Vvi) of each type of structure was calculated according to the relation Vvi = AAi = Ai/A × 100.

### 4.10. Statistical Analysis

An analysis of variance (ANOVA) was applied to the data obtained for the percentage of newly formed bone to verify the effect of the different groups tested in each evaluated period. The homogeneity of variances and normality of residues and the necessary assumptions for the conduction of ANOVA, were tested, respectively, by the Shapiro–Wilk and Bartlett tests, both at 5% probability. Subsequently, the means were compared by the Tukey test at 5% probability. The effect of the period evaluated in each group was compared by Student’s *t*-test at 5%. All analyses were conducted with R (R Core Team, 2017).

## 5. Conclusions

It was concluded that the support system formed by the xenograft fibrin sealant associated with the photobiomodulation therapy protocol had a positive effect on the bone repair process in critical size defects in rat calvaria.

## Figures and Tables

**Table 1:** Therapeutic parameters of the photobiomodulation therapy used in this study.

Parameter	Unit/Explanation
Optical Power	30 mW
Wavelength	830 nm
Density of Power or Irradiance	258.6 mW/cm^2^
Fluency or Density of Energy or Dosimetry	6 J/cm^2^
Beam Area	0.116 cm^2^
Total Power	2.9 J
Type of Beam	Positioned for laser irradiation at perpendicular incidence to the skull
Emission Mode	Continuous (laser power remains constant at all times)
Form of Application	Four points surrounding the surgical area, north, south, east, and west
Duration of Irradiation	24 s/point
Total Time of each Application	96 s
Treatment Time	Immediately after surgery and three times a week until euthanasia

Laser beam emissions were self-calibrated by the device during all applications.
